# Illness Acceptance Among Adults with Melanoma: A Prospective Observational Cross-Sectional Study

**DOI:** 10.3390/cancers18162558

**Published:** 2026-08-09

**Authors:** Paulina Piesch, Maciej Krężel, Hanna Adamska, Inga Buława, Maja Hrynczyszyn, Michalina Świetlicka, Marta Szepietowska, Piotr K. Krajewski, Grażyna Kamińska-Winciorek, Marcin Ziętek, Łukasz Matusiak, Jacek C. Szepietowski

**Affiliations:** 1 Students’ Research Group of Division of Dermatology, Venereology and Clinical Immunology, Faculty of Medicine, Wroclaw University of Science and Technology, 50-377 Wroclaw, Poland; 2 Department of Dermatology, Jagiellonian University Medical College, 31-503 Cracow, Poland; marta.szepietowska@doctoral.uj.edu.pl; 3 Doctoral School of Medical and Health Sciences, Jagiellonian University Medical College, 31-530 Cracow, Poland; 4 Division of Dermatology, Venereology and Clinical Immunology, Faculty of Medicine, Wroclaw University of Science and Technology, 50-377 Wroclaw, Poland; 5 Department of Dermato-Venereology, 4th Military Hospital, 50-981 Wroclaw, Poland; 6 Department of Bone Marrow Transplantation and Haematology-Oncology, Skin Cancer and Melanoma Team, Maria Sklodowska-Curie National Research Institute of Oncology, 44-102 Gliwice, Poland; 7 Lower Silesian Oncology, Pulmonology and Hematology Center, 53-413 Wroclaw, Poland; marcin.zietek@umw.edu.pl; 8 Department of Oncology, Faculty of Medicine, Wroclaw Medical University, 53-413 Wroclaw, Poland

**Keywords:** melanoma, illness acceptance, depression, anxiety, quality of life, psychosocial burden

## Abstract

**Introduction**: Melanoma is a chronic disease that significantly affects patients’ daily functioning and emotional well-being. Beyond its somatic burden, it often leads to substantial changes in quality of life (QoL). This study aimed to assess illness acceptance among melanoma patients and examine its associations with selected clinical characteristics (disease stage, pruritus and time since diagnosis), psychological distress (depression and anxiety) and QoL. **Materials and Methods**: A cross-sectional study was conducted between June 2025 and February 2026 at two tertiary oncology centers in Poland. Initially, 191 consecutive melanoma patients were recruited, of whom 162 (58.6% men; mean age 59.8 ± 13.7 years) were included in the final analysis (response rate: 84.8%). Participants completed validated questionnaires assessing illness acceptance (Acceptance of Illness Scale (AIS)), depression (PHQ-9, HADS-D), anxiety (GAD-7, HADS-A), and QoL (DLQI). Statistical analyses were performed, with *p* < 0.05 considered statistically significant. **Results**: The mean AIS score was 13.28 ± 7.50 points, indicating a remarkably low level of illness acceptance. In the multivariable linear regression analysis, greater depressive symptoms (HADS-D: β = −1.16, 95% CI −1.79 to −0.54; *p* < 0.001) and more advanced melanoma stage (β = −1.52, 95% CI −2.92 to −0.12; *p* = 0.034) were independently associated with lower illness acceptance. In addition, illness acceptance showed significant correlations with depressive symptoms, anxiety symptoms, and dermatology-related QoL, whereas no significant associations were observed with most evaluated demographic and clinical variables. **Conclusions**: Patients with melanoma demonstrated remarkably low illness acceptance. Depressive symptoms and more advanced melanoma stage were independently associated with lower illness acceptance. These findings support the integration of psychological assessment into comprehensive melanoma care.

## Simple Summary

Melanoma affects not only physical health but also the way patients adapt to living with cancer. Psychological adjustment, reflected by illness acceptance, may influence emotional well-being and everyday functioning, yet it has received little attention in melanoma research. In this study, we evaluated illness acceptance in adults with melanoma and explored its relationship with clinical characteristics, symptoms of depression and anxiety, and quality of life. Most patients showed poor illness acceptance. Lower illness acceptance was consistently associated with greater psychological distress and poorer quality of life. Multivariable analysis identified depressive symptoms and more advanced melanoma stage as factors independently associated with lower illness acceptance. These findings suggest that the emotional impact of melanoma is not determined solely by disease severity and that many patients may require psychological support throughout their care. Our findings support the integration of routine psycho-oncological assessment into melanoma care, regardless of disease stage, to promote better patient-centered management and long-term well-being.

## 1. Introduction

The incidence of melanoma has been steadily increasing worldwide, making it one of the most significant public health concerns among skin cancers [1,2]. Melanoma arises from the malignant transformation of melanocytes and is characterized by a high metastatic potential, particularly when diagnosed at advanced stages. Due to its unpredictable course and risk of progression, melanoma is increasingly perceived not only as an oncological condition but also as a chronic, life-threatening disease requiring long-term monitoring and management [3].

Although survival outcomes have substantially improved in recent years owing to earlier detection and the introduction of modern systemic therapies, many patients continue to live with persistent uncertainty regarding disease recurrence, progression, and the need for ongoing medical follow-up. Consequently, melanoma should be viewed not only as an acute oncological condition but also as a chronic disease with long-term medical and psychosocial consequences [3,4,5].

Against this background, the psychological burden of melanoma has become increasingly recognized [4,5,6]. The necessity for regular dermatological follow-up, the risk of recurrence, and—in more advanced stages—the need for systemic therapies such as immunotherapy or targeted treatment contribute to a persistent sense of uncertainty and threat [1,4,7]. These factors may lead to emotional distress, including symptoms of depression and anxiety, and significantly impair quality of life (QoL).

In the context of chronic disease, psychological adaptation plays a crucial role in overall patient well-being. One of the key components of such adaptation is illness acceptance, understood as the ability to integrate the disease into one’s life while minimizing its negative emotional and functional consequences. Higher levels of illness acceptance are associated with better coping strategies, lower psychological distress, and improved QoL, as demonstrated in various chronic conditions, including dermatological diseases [8,9].

The Acceptance of Illness Scale (AIS) is a widely used tool for assessing the degree of psychological adaptation to chronic illness. It reflects the subjective perception of disease-related limitations, dependence, and emotional burden [10]. Although depression, anxiety, fear of cancer recurrence, and QoL have been increasingly investigated in melanoma, psychological adaptation measured as illness acceptance remains largely unexplored [5,6]. Based on previous evidence demonstrating substantial psychological burden among patients with melanoma, we hypothesized that illness acceptance would be low and associated with psychological distress and impaired QoL rather than with objective clinical characteristics. Therefore, the primary aim of this study was to evaluate the level of illness acceptance among patients with melanoma. Additionally, we sought to investigate the relationship between illness acceptance and selected clinical factors, including disease stage, pruritus, and time since diagnosis, as well as psychological distress (depression and anxiety) and dermatology-related QoL.

## 2. Materials and Methods

### 2.1. Study Design and Participants

A cross-sectional observational study was conducted between June 2025 and February 2026. Consecutive adult patients with histologically confirmed melanoma were recruited during routine outpatient visits at two tertiary oncology centers in Poland: the Maria Skłodowska-Curie National Research Institute of Oncology, Gliwice Branch, and the Lower Silesian Oncology, Pulmonology and Hematology Center in Wroclaw, Poland. Patients were invited to participate by the treating physicians during scheduled follow-up appointments. Initially, 191 individuals were recruited, of whom 162 were included in the final analysis (response rate: 84.8%). Demographic and clinical data were collected using a structured questionnaire. This included age, gender, time since diagnosis, disease stage, presence and intensity of pruritus. The clinical stage of melanoma was determined based on the American Joint Committee on Cancer (AJCC) classification [11]. The worst intensity of pruritus during the disease course was assessed with Numeral Rating Scale (NRS) [12].

The final study group consisted of 67 (41.4%) females and 95 (58.6%) males. The mean age of participants was 59.8 ± 13.7 years (range: 29–92 years). The clinical characteristics of the cohort reflected substantial heterogeneity in disease progression. Patients were distributed across all stages of melanoma according to the AJCC classification, with the largest subgroups represented by stage II (29.0%) and stage IV (25.9%) disease. The mean time since diagnosis was 101.9 ± 316.4 weeks, indicating considerable variability between recently diagnosed patients and long-term survivors. Among the physical symptoms, pruritus was the only symptom specifically evaluated in this study, as it was considered a clinically relevant parameter for the present analysis. Pruritus was reported by 24.1% of patients. However, its overall intensity was low, with a mean score of 0.9 ± 1.9 points. Detailed demographic and clinical characteristics of the study population are presented in Table 1.

Participants were asked to complete a set of standardized, validated questionnaires assessing psychological functioning and QoL. Participation in the study was voluntary and anonymous. All participants provided informed consent prior to inclusion. The study protocol was approved by the local Ethics Committee of the Lower Silesian Chamber of Physicians and Dentists (decision number—13/BNBO/2025).

### 2.2. Psychometric Assessments

The following validated instruments were used:

#### 2.2.1. Acceptance of Illness Scale (AIS)

The AIS was used to evaluate the degree of psychological adaptation to chronic illness. The scale, originally developed by Felton, Revenson, and Hinrichsen, is based on the assumption that chronic disease is associated with various limitations, including reduced independence, decreased self-esteem, and increased dependence on others [10]. The AIS consists of 8 statements referring to the negative consequences of illness. Each item is rated on a 5-point Likert scale ranging from 1 (“strongly agree”) to 5 (“strongly disagree”). The total score ranges from 8 to 40 points, with higher scores indicating greater illness acceptance and better psychological adaptation [10]. According to standardized interpretation guidelines (Polish adaptation by Juczyński [13]), AIS scores are categorized as follows: <20 points—low level of illness acceptance (poor adaptation, significant psychological distress); 20–30 points—moderate level of acceptance; and >30 points—high or full acceptance of the disease. This classification allows for the identification of patients at risk of maladaptive coping and increased psychological burden.

#### 2.2.2. Dermatology Life Quality Index (DLQI)

The DLQI was used to assess the impact of skin disease on patients’ QoL over the previous week. The questionnaire consists of 10 items covering symptoms and feelings, daily activities, leisure, work and school, personal relationships, and treatment. Each item is scored from 0 to 3 points, yielding a total score ranging from 0 to 30 points, with higher scores indicating greater impairment in quality of life. The DLQI is one of the most widely used dermatology-specific QoL instruments, allowing comparison across different skin diseases [14].

#### 2.2.3. Hospital Anxiety and Depression Scale (HADS)

The HADS was used to assess symptoms of anxiety and depression in patients with somatic diseases. The scale was specifically designed to minimize the influence of physical symptoms of illness on psychological assessment. The questionnaire consists of 14 items divided into two subscales: HADS-A (anxiety)—7 items and HADS-D (depression)—7 items. Each item is scored from 0 to 3 points, resulting in subscale scores ranging from 0 to 21 points. Higher scores indicate greater severity of anxiety (HADS-A) or depressive symptoms (HADS-D). The use of HADS is particularly justified in medical populations, as it excludes somatic symptoms such as fatigue or sleep disturbances, which may overlap with symptoms of chronic disease [15].

#### 2.2.4. Patient Health Questionnaire-9 (PHQ-9)

PHQ-9 was used to assess the severity of depressive symptoms based on the Diagnostic and Statistical Manual of Mental Disorders (DMS) diagnostic criteria for major depressive disorder. The questionnaire consists of 9 items, each scored from 0 (“not at all”) to 3 (“nearly every day”), resulting in a total score ranging from 0 to 27 points. Higher PHQ-9 scores indicate greater severity of depressive symptoms, with higher values reflecting a greater likelihood of clinically relevant depression [16,17].

#### 2.2.5. Generalized Anxiety Disorder-7 (GAD-7)

GAD-7 was used to evaluate the severity of anxiety symptoms over the previous two weeks. The questionnaire includes 7 items rated on a 4-point Likert scale from 0 (“not at all”) to 3 (“nearly every day”), with total scores ranging from 0 to 21 points. Higher GAD-7 scores indicate greater severity of anxiety symptoms [18,19].

The use of multiple instruments to assess depression and anxiety (HADS, PHQ-9, and GAD-7) was intentional and aimed to provide a comprehensive evaluation of psychological distress. While PHQ-9 and GAD-7 are based on DSM diagnostic criteria and allow for grading symptom severity, HADS is specifically designed for patients with somatic diseases and minimizes the confounding effect of physical symptoms. This combined approach enables a more robust and multidimensional assessment of mental health in patients with melanoma.

### 2.3. Statistical Analysis

Statistical analyses were performed using IBM SPSS Statistics (version 26.0). The normality of data distribution was assessed using the Shapiro–Wilk test. Descriptive statistics are presented as means, standard deviations (SDs), and ranges. Comparisons between groups were conducted using Student’s *t*-test or the Mann–Whitney U test, depending on data distribution. For comparisons involving more than two groups, one-way ANOVA or the Kruskal–Wallis test was applied. Correlations between variables were assessed using Pearson’s or Spearman’s correlation coefficients, as appropriate. Categorical variables were analyzed using the chi-square (χ^2^) test.

To identify factors independently associated with illness acceptance, a multiple linear regression was performed with the AIS total score as the dependent variable. Sociodemographic, clinical, and psychological variables were entered simultaneously as predictors: sex, AJCC stage, presence of pruritus, depression (HADS-D), anxiety (HADS-A), and QoL (DLQI). Unstandardized regression coefficients (β) with 95% confidence intervals (CIs) were reported. Multicollinearity was assessed using variance inflation factors (VIFs), and the assumptions of linearity, homoscedasticity, and normality of residuals were verified through inspection of residual plots. A two-tailed *p*-value of ≤0.05 was considered statistically significant.

## 3. Results

### 3.1. Illness Acceptance in Relation to Demographic and Clinical Data

**Figure 1:**
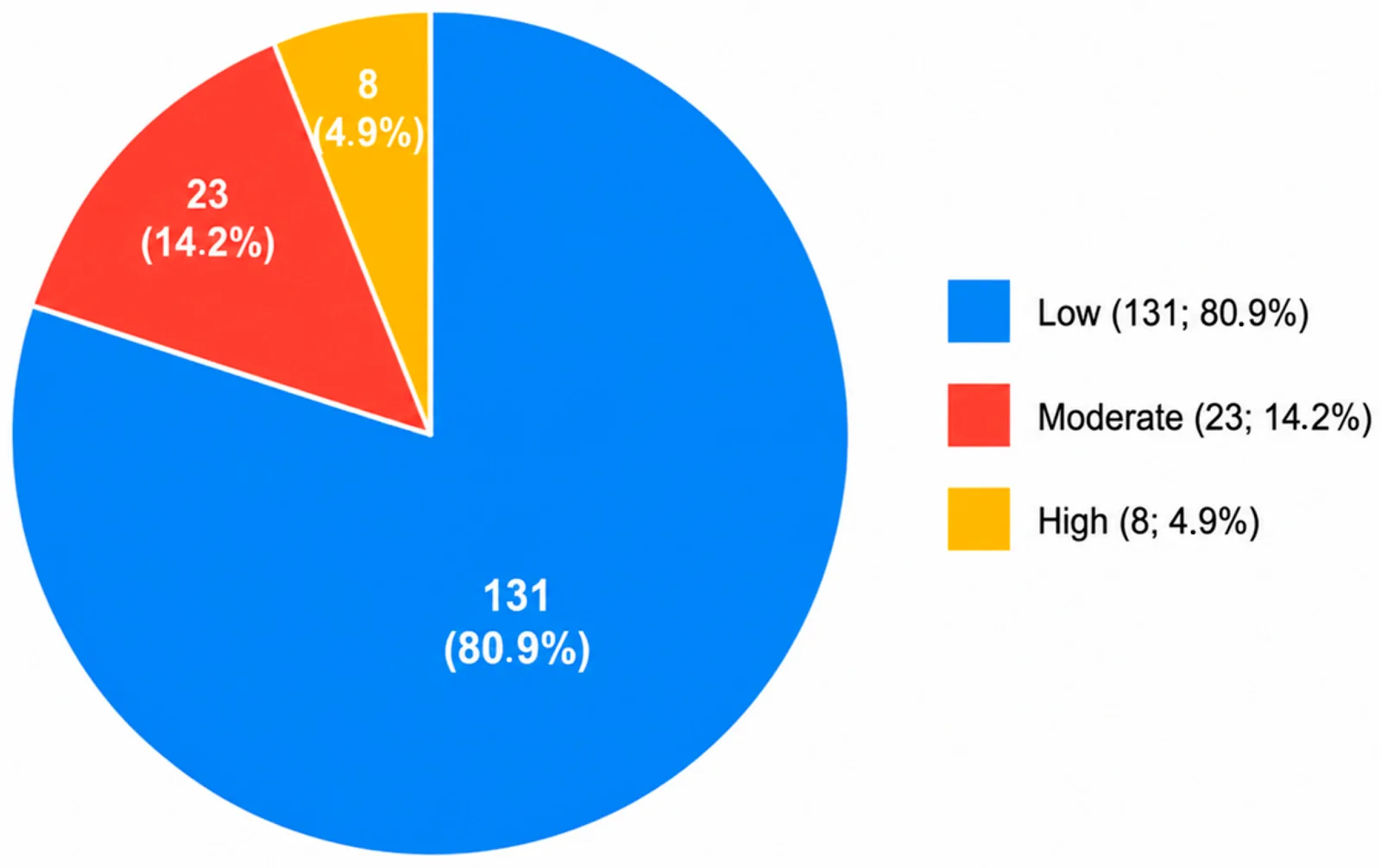
Level of illness acceptance.

The study cohort consisted of 162 patients whose demographic and clinical characteristics are summarized in Table 1. The mean AIS score in the study group was 13.28 ± 7.50 points (range: 0–40 points). According to standardized cut-off values, the vast majority of participants (80.9%, n = 131) demonstrated a low level of illness acceptance. Moderate acceptance was observed in 14.2% (n = 23), while only 4.9% (n = 8) of patients presented high levels of acceptance (Figure 1).

Illness acceptance was not significantly associated with demographic or clinical variables. No significant gender differences were observed (*p* = 0.343), with comparable mean AIS scores in men (12.96 ± 7.35 points) and women (13.73 ± 7.74 points). Similarly, melanoma stage (AJCC) did not significantly influence illness acceptance (*p* = 0.290). Although patients with stage IV disease exhibited slightly higher mean AIS scores (15.19 ± 9.97 points) compared to those with stage 0 (9.00 ± 1.73 points), these differences did not reach statistical significance. Furthermore, the presence of pruritus had no significant effect on illness acceptance (*p* = 0.752). No significant correlations were found between illness acceptance and clinical parameters, including pruritus intensity and time since the diagnosis (*p* > 0.05 for all comparisons).

### 3.2. Correlations Between Illness Acceptance and Psychological and Clinical Factors

Correlation analyses revealed statistically significant, moderate negative associations between illness acceptance and all assessed psychological variables. Higher levels of depressive symptoms were associated with lower illness acceptance, as demonstrated by both PHQ-9 (r = −0.387, *p* < 0.001) and HADS-D (r = −0.405, *p* < 0.001). Similarly, anxiety measures showed negative correlations with AIS scores (GAD-7: r = −0.325, *p* < 0.001 and HADS-A: r = −0.327, *p* < 0.001, respectively). A comparable relationship was observed for QoL, with DLQI scores negatively correlating with illness acceptance (r = −0.369, *p* < 0.001), indicating that greater QoL impairment is associated with poorer psychological adaptation to the disease. Detailed correlation coefficients are presented in Table 2.

### 3.3. Multiple Linear Regression Analysis

In the multiple linear regression with the AIS total score as the dependent variable, the model was statistically significant and explained 22% of the variance in illness acceptance (adjusted R^2^ = 0.17; F (10,139) = 4.00; *p* < 0.001) (Table 3). After simultaneous adjustment for sociodemographic, clinical, and psychological variables, lower illness acceptance was independently predicted by more severe depressive symptoms (HADS-D: β = −1.16; 95% CI −1.79 to −0.54; *p* < 0.001) and more advanced melanoma stage (β = −1.52; 95% CI −2.92 to −0.12; *p* = 0.034). Anxiety (HADS-A), QoL (DLQI), sex and pruritus were not independently associated with illness acceptance. Notably, AJCC stage, which showed no significant univariate association with illness acceptance, emerged as an independent predictor in the multivariable model.

## 4. Discussion

Illness acceptance constitutes an important component of psychological adaptation to chronic disease [8,9]. Rather than reflecting resignation, it describes the process through which individuals integrate the illness into their lives while preserving a sense of autonomy, self-worth, and personal identity. This multidimensional construct encompasses cognitive, emotional, and behavioral adaptation to disease-related limitations and has consistently been associated with more effective coping strategies, lower emotional distress, greater treatment adherence, and better overall QoL across numerous chronic medical conditions [20]. Importantly, illness acceptance should be viewed as a dynamic process that evolves throughout the disease course and may fluctuate depending on disease progression, treatment experiences, perceived prognosis, and changing life circumstances [21,22]. Consequently, evaluating illness acceptance provides valuable insight into patients’ psychological functioning beyond conventional clinical indicators.

Although illness acceptance has been investigated in several chronic dermatological disorders, evidence regarding melanoma remains remarkably limited. Most psycho-oncological studies in melanoma have focused on depression, anxiety, fear of recurrence, distress, or QoL, whereas psychological adaptation measured using standardized illness acceptance instruments has received considerably less attention [22]. This lack of evidence is surprising because melanoma differs fundamentally from most inflammatory skin diseases [2]. Beyond visible skin lesions, patients must cope with the awareness of a potentially life-threatening malignancy, uncertainty regarding recurrence or progression, prolonged surveillance, and, in many cases, repeated diagnostic and therapeutic procedures [4,7]. These unique characteristics may substantially influence the process of illness acceptance.

Comparison with previous dermatological studies further emphasizes the distinctive psychological burden associated with melanoma [23]. Young adults with acne generally demonstrate high illness acceptance, while patients with psoriasis usually present moderate levels that are influenced primarily by symptom severity, particularly pruritus, and QoL impairment [24,25,26]. In contrast, the exceptionally low AIS scores observed in our cohort indicate substantially poorer psychological adaptation. Unlike inflammatory dermatoses, melanoma introduces a persistent perception of existential threat, which may remain present even after apparently successful treatment. Therefore, the psychological burden associated with melanoma appears to extend beyond physical symptoms alone and reflects the profound impact of a cancer diagnosis on patients’ perception of their future, health, and personal security [4,5,27].

The multivariable analysis provided additional insight into the determinants of illness acceptance. Although melanoma stage was not significantly associated with illness acceptance in the univariate analyses, it emerged as an independent predictor after adjustment for demographic, clinical, and psychological variables. This finding suggests that the psychological impact of disease severity may become more apparent after controlling for coexisting emotional factors. Patients with more advanced melanoma are exposed to greater treatment burden, increased uncertainty regarding prognosis, and a higher perceived risk of disease progression, all of which may adversely affect psychological adaptation despite the absence of a simple univariate relationship [5,27]. Similar observations have been reported in psycho-oncological studies indicating that objective disease burden and subjective psychological adaptation are not always linearly related but may interact with emotional and psychosocial factors [5].

Illness acceptance demonstrated consistent associations with patients’ psychological functioning. Importantly, these associations were consistently observed across two different psychometric approaches to the assessment of depression and anxiety, which strengthens the robustness of the present findings. Lower AIS scores correlated with greater depressive symptoms, higher anxiety levels, and poorer dermatology-related QoL. Depression appeared an independent predictor factor for lower illness acceptance in multiple linear regression analysis. These observations are consistent with studies conducted in both dermatological and non-dermatological chronic diseases, suggesting that illness acceptance represents a central determinant of emotional adjustment [24,25,26,28]. Individuals who successfully integrate the disease into their daily lives appear better able to cope with uncertainty and maintain psychological well-being, whereas poor acceptance may perpetuate maladaptive coping strategies, emotional distress, and functional impairment [21]. Although the cross-sectional design precludes causal inference, the observed relationships highlight the close interaction between illness acceptance and psychological health.

From a clinical perspective, our findings suggest that assessment of psychological adaptation may represent a valuable component of comprehensive melanoma care. This recommendation is supported by the remarkably high proportion of patients demonstrating poor illness acceptance (80.9%), together with the consistent associations observed between illness acceptance, depression, anxiety, and impaired quality of life. Although further prospective studies are warranted, these findings indicate that psychological adaptation deserves greater attention during routine melanoma follow-up. Since illness acceptance was unrelated to objective disease stage but closely associated with depression, anxiety, and QoL, clinicians should not assume that patients with apparently favorable prognoses experience minimal psychological burden. Routine psycho-oncological screening, timely psychological consultation, patient education, and interventions aimed at strengthening adaptive coping strategies may facilitate adjustment to the diagnosis and improve overall well-being. Such supportive measures should be considered throughout the disease course rather than being reserved exclusively for patients with advanced melanoma.

Several limitations of this study should be acknowledged. First, the cross-sectional design precludes conclusions regarding causal relationships between illness acceptance and psychological outcomes. Longitudinal studies are needed to determine how illness acceptance evolves throughout different stages of melanoma management and survivorship. Second, all psychological variables were assessed using self-administered questionnaires. Although validated instruments were employed, self-reported measures remain susceptible to recall and response bias. Furthermore, several potentially important determinants of illness acceptance, including coping styles, resilience, personality characteristics, social support, fear of cancer recurrence, and previous psychiatric treatment, were not evaluated. Additionally, potentially relevant sociodemographic characteristics (e.g., place of residence, educational level, marital or employment status) and non-melanoma comorbidities were not collected and therefore could not be evaluated as determinants of illness acceptance. Finally, despite enrolling patients across all AJCC stages, subgroup sizes were relatively limited, which may have reduced statistical power for detecting subtle differences between clinical stages.

## 5. Conclusions

To our knowledge, this is the first study to comprehensively evaluate illness acceptance among patients with melanoma and its associations with clinical characteristics, psychological distress, and dermatology-related QoL. Patients with melanoma demonstrated remarkably low illness acceptance, which was consistently associated with greater depressive and anxiety symptoms and poorer QoL. Moreover, multivariable analysis identified depressive symptoms and more advanced melanoma stage as factors independently associated with lower illness acceptance. These findings underscore the importance of psychological adaptation in melanoma and support the incorporation of psychosocial assessment into comprehensive patient care. Future prospective studies should determine whether interventions aimed at improving psychological well-being can enhance illness acceptance and long-term patient outcomes.

## Figures and Tables

**Table 1:** Group characteristics.

Characteristics	Details	
Gender N (%)	Female	67 (41.4%)
	Male	95 (58.6%)
Age [years] (Mean ± SD)	59.8 ± 13.7	
Lesion duration [days] (Mean ± SD)	101.9 ± 316.4	
Pruritus N (%)	Yes	39 (24.1%)
	No	123 (75.9%)
Pruritus intensity [points] (NRS)	0.9 ± 1.9	
Melanoma stage AJCC N (%)	0	3 (1.9%)
	I	33 (20.4%)
	II	47 (29.0%)
	III	37 (22.8%)
	IV	42 (25.9%)

N—number, SD—standard deviation, NRS—Numeral Rating Scale, AJCC—American Joint Committee on Cancer.

**Table 2:** Correlation coefficients (r) between Acceptance of Illness Scale (AIS) and psychological and clinical variables.

Variable	Correlation Coefficient (r)	*p*-Value
Psychological Factors		
Depression (PHQ-9)	−0.387	<0.001
Depression (HADS-D)	−0.405	<0.001
Anxiety (GAD-7)	−0.325	<0.001
Anxiety (HADS-A)	−0.327	<0.001
Quality of Life (DLQI)	−0.369	<0.001
Clinical Parameters		
Time since diagnosis [weeks]	0.008	0.921
Pruritus intensity (NRS) [points]	0.033	0.690

PHQ-9—Patient Health Questionnaire; HADS-D—Hospital Anxiety and Depression Scale—Depression, GAD-7—Generalized Anxiety Disorder-7, HADS-A—Hospital Anxiety and Depression Scale—Anxiety, DLQI—Dermatology Life Quality Index, NRS—Numeral Rating Scale.

**Table 3:** Multiple linear regression—dependent variable: Acceptance of Illness Scale (AIS) total score.

Predictor	β	95% CI	*p*
Sex (male)	0.57	−2.60–3.73	0.724
AJCC stage	−1.52	−2.92–−0.12	0.034
Pruritus (presence)	2.01	−1.69–5.71	0.285
Depression (HADS-D)	−1.16	−1.79–−0.54	<0.001
Anxiety (HADS-A)	0.28	−0.32–0.88	0.352
Quality of Life (DLQI)	−0.18	−0.67–0.32	0.479

Model R^2^ = 0.22; adjusted R^2^ = 0.17; F (10,139) = 4.00; *p* < 0.001. All variance inflation factors (VIFs) < 2.5. β—unstandardized regression coefficient; CI—confidence interval; HADS-D/HADS-A—Hospital Anxiety and Depression Scale (depression/anxiety); DLQI—Dermatology Life Quality Index.

## Data Availability

The datasets generated and/or analyzed during the current study are available from the corresponding author upon reasonable request. The datasets are not publicly available because they contain data derived from human participants and form part of an ongoing research project, for which additional analyses and publications are planned. Requests for access to the data will be considered on a case-by-case basis, subject to institutional approval, participant confidentiality, and applicable ethical and legal requirements.

## Abbreviations

The following abbreviations are used in this manuscript:

AIS	Acceptance of Illness Scale
AJCC	American Join Committee on Cancer
DLQI	Dermatology Life Quality Index
DSM	Diagnostic and Statistical Manual of Mental Disorders
GAD-7	Generalized Anxiety Disorder-7
HADS	Hospital Anxiety and Depression Scale
HADS-A	Hospital Anxiety and Depression Scale—Anxiety
HADS-D	Hospital Anxiety and Depression Scale—Depression
N	Number
NRS	Numerical Rating Scale
PHQ-9	Patient Health Questionnaire-9
QoL	Quality of Life
SD	Standard Deviation
